# Supplementary material for: More than Just a Number: Perspectives from Black Male Participants on Community-Based Interventions and Clinical Trials to Address Cardiovascular Health Disparities
Source: Int J Environ Res Public Health. 2024 Apr 6;21(4):449. doi: 10.3390/ijerph21040449 (PMC11050149; doi:10.3390/ijerph21040449)
Supplement: Supplementary file 1 [file ijerph-21-00449-s001.zip › ijerph-2887406-supplementary.pdf]

Focus Group Questions

Enrollment in clinical trials

1. How, if at all, did participation in the study change your view on clinical trials?
    a. Gained more or less trust in clinical trials? Increased knowledge on clinical trial participation?
    b. Impact of Black health coaches
2. What drew you into participating with this study?
    a. Were the incentives persuasive?
3. How can we increase participation with surveys?
    a. Participation with surveys would of changed if a session for just completing surveys was offered?
